# Tracking the Evolving Role of Artificial Intelligence in Implementation Science: Protocol for a Living Scoping Review of Applications, Evaluation Approaches and Outcomes

**DOI:** 10.12688/f1000research.171774.2

**Published:** 2026-02-12

**Authors:** Guillaume Fontaine, Olivia Di Lalla, Susan Michie, Byron J. Powell, Vivian Welch, James Thomas, Jeffery Chan, Samira Abbasgholizadeh-Rahimi, France Légaré, Janna Hastings, Sylvie D. Lambert, Justin Presseau, Sharon E. Straus, Ian D. Graham, Ruopeng An, Daniel N. Elakpa, Meagan Mooney, Alenda Dwiadila Matra Putra, Rachael Laritz, Natalie Taylor

**Affiliations:** 1 McGill University Ingram School of Nursing, Montreal, Québec, Canada; 2 Lady Davis Institute for Medical Research Centre for Clinical Epidemiology, Montreal, Québec, Canada; 3 Centre for Implementation Research, Ottawa Hospital Research Institute, Ottawa, Ontario, Canada; 4 University College London Centre for Behaviour Change, London, England, UK; 5 Brown School, Washington University in St Louis George Warren Brown School of Social Work, St. Louis, Missouri, USA; 6 University of Ottawa School of Epidemiology and Public Health, Ottawa, Ontario, Canada; 7 Bruyère Health Research Institute, Ottawa, Canada; 8 University College London Social Research Institute, London, England, UK; 9 Institute of Education, University College London, London, England, UK; 10 School of Population Health, UNSW Sydney, University of New South Wales, Sydney, Australia; 11 Department of Family Medicine, McGill University, Montreal, Québec, Canada; 12 Mila – Quebec Artificial Intelligence Institute, Montreal, Canada; 13 Faculty of Dental Medicine and Oral Health Sciences, McGill University, Montreal, Québec, Canada; 14 Department of Family and Emergency Medicine, Universite Laval, Québec City, Québec, Canada; 15 VITAM Research Centre for Sustainable Health, Quebec City, Canada; 16 Institute for Implementation Science in Health Care, Universitat Zurich, Zürich, Zurich, Switzerland; 17 University of St Gallen School of Medicine, St. Gallen, St. Gallen, Switzerland; 18 Swiss Institute of Bioinformatics, Lausanne, Vaud, Switzerland; 19 CIUSSS West-Montreal, St Mary's Research Centre, Montreal, Québec, Canada; 20 University of Ottawa School of Psychology, Ottawa, Ontario, Canada; 21 Unity Health Toronto, St Michael's Hospital Li Ka Shing Knowledge Institute, Toronto, Ontario, Canada; 22 University of Toronto Institute of Health Policy Management and Evaluation, Toronto, Ontario, Canada; 23 University of Toronto Department of Medicine, Toronto, Ontario, Canada; 24 New York University Silver School of Social Work, New York, New York, USA; 25 CIUSSS West-Central Montreal, Jewish General Hospital, Montreal, Canada

**Keywords:** machine learning; deep learning; natural language processing; large language models; generative AI; ChatGPT; sentiment analysis; implementation research; decision support; health systems

## Abstract

**Background:**

Artificial intelligence (AI) offers significant opportunities to improve the field of implementation science by supporting key activities such as evidence synthesis, contextual analysis, and decision-making to promote the adoption and sustainability of evidence-based practices. This living scoping review aims to: (1) map applications of AI in implementation research and practice; (2) identify evaluation approaches, reported outcomes, and potential risks; and (3) synthesize reported research gaps and opportunities for advancing the use of AI in implementation science.

**Methods:**

This scoping review will follow the Joanna Briggs Institute (JBI) methodology and the Cochrane guidance for living systematic reviews. A living scoping review is warranted to keep up with the rapid changes in AI and its growing use in implementation science. We will include empirical studies, systematic reviews, grey literature, and policy documents that describe or evaluate applications of AI to support implementation science across the steps of the Knowledge-to-Action (KTA) Model. AI methods and models of interest include machine learning, deep learning, natural language processing, large language models, and related technologies and approaches. A search strategy will be applied to bibliographic databases (MEDLINE, Embase, CINAHL, PsycINFO, IEEE Xplore, Web of Science), relevant journals, conference proceedings, and preprint servers. Two reviewers will independently screen studies and extract data on AI characteristics, specific implementation task according to the KTA Model, evaluation methods, outcome domains, risks, and research gaps. Extracted data will be analyzed descriptively and synthesized narratively using a mapping approach aligned with the KTA Model.

**Discussion:**

This living review will consolidate the evidence base on how AI is applied across the spectrum of implementation science. It will inform researchers, policymakers, and practitioners seeking to harness AI to improve the adoption, scale-up, and sustainability of evidence-based interventions, while identifying areas for methodological advancement and risk mitigation.

**Review registration:**

Open Science Framework, May 2025: https://doi.org/10.17605/OSF.IO/2Q5DV

## Introduction

Health systems grapple with the overuse of harmful, wasteful, or ineffective interventions, commonly referred to as “low-value care,” while underutilizing evidence-based interventions, leading to gaps in the delivery of “high-value care.” ^1, 2^ Implementation science, defined as the study of methods and strategies that facilitate the integration of evidence-based interventions, programs, and policies into health systems, ^3^ holds considerable promise in addressing these challenges across a range of clinical contexts. ^4– 10^ It seeks to understand how, why, and under what conditions implementation succeeds or fails across varying contexts, and how best to support healthcare provider behaviour and system change. ^11, 12^ Implementation research refers to the rigorous investigation of these methods and strategies, while implementation practice concerns their application by practitioners, health system leaders, and policymakers in real-world settings. ^13^ However, implementation efforts to adopt and sustain evidence-based interventions are constrained by the time- and resource-intensive processes required to identify, synthesize, and apply implementation evidence, including context-specific barriers, facilitators, and strategies. ^14, 15^ Additional challenges, including the heterogeneity of implementation data, variability in outcomes, and the underrepresentation of key populations, hinder timely, equitable, and context-responsive implementation. ^16^ These limitations also undermine sustainability. ^17^

In recent years, the integration of artificial intelligence (AI) in science and practice has rapidly advanced across sectors. ^18^ AI has long been implemented in healthcare across diagnostics, treatment, population health management, patient care, and healthcare professional training and decision support, utilizing a wide range of AI innovations. ^19, 20^ It is helpful to distinguish between the major categories of AI and the methods that power them. Machine learning (ML) is a foundational approach in which systems learn patterns from data, with deep learning (DL) being a specialized subset that uses multi-layered neural networks for complex pattern recognition. Natural language processing (NLP) is a field of AI focused on enabling machines to understand and generate human language, often powered by DL-based models such as large language models (LLMs). ^19, 20^ LLMs are also an example of generative AI, which encompasses models capable of creating new content, such as text or images. ^21– 23^

Most healthcare-related systems use ML, DL and generative AI methods. ^19^ ML and DL models have improved diagnostic accuracy by analyzing medical images and large datasets, reducing human error in disease detection ^24, 25^; for example, in breast cancer screening, they can lower both false positives and false negatives. ^26, 27^ ML-based methods are advancing personalized medicine, particularly in oncology, where it aids in genomic analysis to predict drug responses and disease predispositions. ^28– 30^ ML-driven predictive analytics support population health management by identifying at-risk individuals and enabling early interventions, thereby reducing hospital readmissions and healthcare costs. ^31^ Virtual assistants powered by NLP are automating routine tasks, providing continuous support, and even offering mental health support through web-based cognitive-behavioral therapy. ^32, 33^

The recent momentum in AI-driven healthcare is largely propelled by advancements in generative AI in the form of LLMs, such as Open AI’s GPT, ^21^ Meta AI’s LLaMA ^22^ and Google DeepMind’s Gemma, ^23^ which introduce new possibilities in medical documentation, patient risk assessment, and clinical decision-making. ^34^ LLMs are AI models trained on vast amounts of textual data to process, understand, and generate human-like language. ^21– 23^ These models are based on deep learning architectures, such as transformers, which allow them to analyze and predict text patterns effectively. ^34^ Their applications are wide-ranging; for example, it is claimed that they can assist healthcare professionals by summarizing clinical encounters, automating medical notetaking, and generating real-time responses to complex medical queries, providing support that can increase efficiency and allow healthcare professionals to focus more on patient care. ^34^ They may also contribute to the education and training of health professionals and patients. ^35, 36^ LLMs like ChatGPT may benefit medical education by supporting differential diagnosis brainstorming and providing interactive clinical cases for practice. ^34^ LLMs have also shown effectiveness in patient education by delivering accurate answers to questions, enriching and tailoring existing educational resources, and simplifying complex medical language into more accessible terms. ^35, 36^

**Figure 1.**
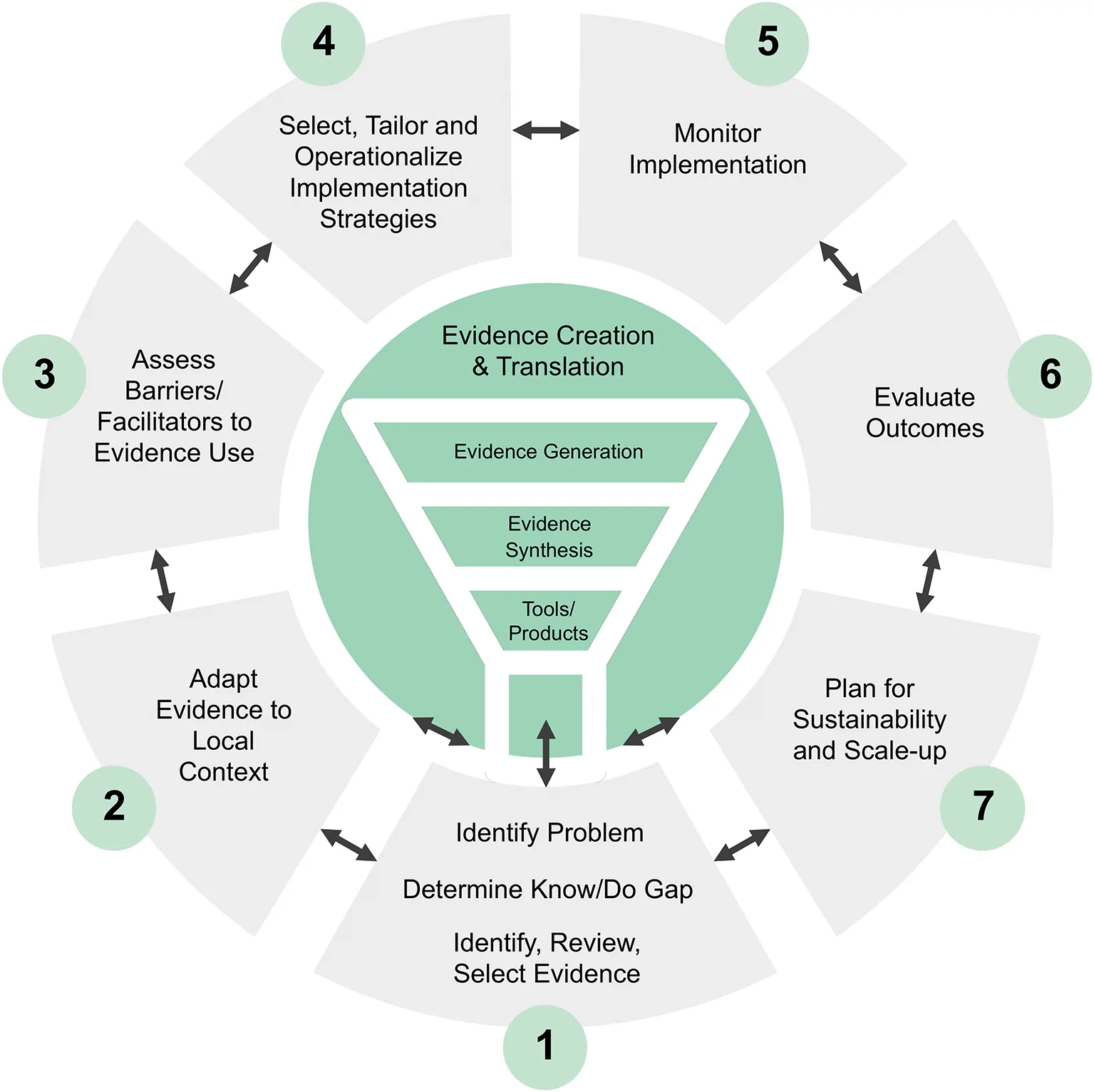
Knowledge to action model, adapted from Graham et al. ^38^

Given this momentum, interest in harnessing AI for implementation research and practice is rapidly growing. ^37^ AI offers new opportunities to improve the speed and efficiency of all steps of the Knowledge-to-Action (KTA) Model, ^38^ from conducting the synthesis of implementation evidence to planning for sustainability and scale-up (see Figure 1). Recent advances in AI-driven evidence synthesis and decision-making support for human behavior change and implementation science ^39, 40^ are exemplified by the Human Behaviour-Change Project (HBCP), which employs AI and ML to extract, synthesize, interpret, and predict findings from behavior change interventions, thereby guiding practitioners, policymakers, and researchers on what works, for whom, under which conditions. ^39, 41, 42^ AI has also been leveraged to explore contextual factors influencing clinician adherence to guidelines. ^43^ Additionally, NLP has been applied to qualitative data analysis, identifying codes and major themes. ^42, 44, 45^ Overall, AI can help address critical challenges in implementation science by enabling rapid evidence synthesis, enhancing data analysis, supporting complex decision-making, and improving the translation of evidence into practice.

Despite its potential, AI remains susceptible to a range of ethical, clinical, technical, and environmental risks. Ethically, algorithmic bias can perpetuate or exacerbate health disparities when training datasets underrepresent certain populations, while data privacy concerns regarding the storage and sharing of sensitive patient data also poses ethical challenges. ^46, 47^ AI’s “black box” nature also complicates interpretability and accountability, posing challenges for both clinicians and regulators. ^48^ Clinically, overdiagnosis and overtreatment may occur when AI systems identify ambiguous findings or produce erroneous recommendations. ^46, 47^ Technically, LLMs and other DL algorithms can generate “hallucinations”. ^34^ While all outputs generated by these algorithms are synthetic and probabilistic constructions, “hallucinations” refer to outputs that are convincing but factually inaccurate, which may mislead healthcare providers. ^34^ Finally, from an environmental perspective, energy-intensive AI training and deployment contribute to a sizeable carbon footprint. ^49^ Addressing these intersecting risks will be essential to harness AI’s benefits while mitigating potential harms in the context of implementation research and practice.

To address the need to track and map quickly evolving fields, authors have proposed a living scoping review approach. ^50^ A living scoping review is a scoping review that is kept continually up to date through ongoing, planned searches and screening at regular intervals, with new eligible evidence incorporated as it appears and the public record refreshed (e.g., updated results, tables, figures, and versioned updates) so the map of the literature remains current. ^50^ This living scoping review aims to provide a comprehensive mapping of the applications of AI relevant to implementation research and practice. The findings will offer a foundation for guidance on harnessing AI to accelerate the adoption of evidence-based practices in healthcare.

### Objectives

The primary objective of this living scoping review is to systematically map and characterize how AI is used to support implementation research and practice. Specifically, we will:

1. Map applications of AI across implementation research and practice activities, including their features, characteristics and implementation contexts, using the KTA Model as an organizing framework.
2. Identify the evaluation approaches, outcomes, and risks reported in AI-enabled implementation research and practice, with attention to technical performance, equity considerations, and unintended consequences.
3. Synthesize evidence gaps and future directions for advancing the responsible and equitable use of AI in implementation science.

A secondary objective is to maintain an up-to-date evidence base using a living scoping review approach, enabling continuous integration of new findings in this fast-evolving field.

## Methods

### Scoping review design

The proposed review will be conducted following the Joanna Briggs Institute (JBI) methodology for scoping reviews, ^51^ and Cochrane’s guidance for living systematic reviews. ^52^ This topic lends itself to a living scoping review approach for several reasons. First, AI technologies and methods are evolving at an unprecedented pace, leading to rapid shifts in the evidence base. Second, implementation science is inherently dynamic and context-specific, necessitating regular updates to capture emerging data, methods, and applications. Third, bridging AI and implementation science is still an emerging area, and a living review ensures that newly published insights are promptly synthesized and integrated. Finally, maintaining an up-to-date map of AI’s potential contributions to implementation science can guide researchers, policymakers, and practitioners as they refine methodologies, prioritize resource allocation, and incorporate novel AI tools into practice.

This protocol addresses the first three steps of JBI’s nine-step approach: first, defining the review objectives and questions; second, developing and aligning the inclusion criteria with these objectives and questions; and third, specifying the methods for evidence searching, selection, data extraction, and presentation. The next four steps will involve evidence searching, selection, extraction, and analysis. The eighth step focuses on presenting the results, while the ninth and final step involves summarizing the evidence, drawing conclusions, and discussing the implications of the findings. ^51^ The reporting of the scoping review will follow the Preferred Reporting Items for Systematic Reviews and Meta-Analyses for Scoping Reviews (PRISMA-ScR) ^53^ and its extension for living systematic reviews (PRISMA-LSR). ^54^

### Review team

Our interdisciplinary team brings extensive, internationally recognized expertise in implementation science, AI, behavioral science, and evidence synthesis. Team members have led or contributed to major advancements in the use of AI in implementation science. For example, Susan Michie and Janna Hastings have been central to the *Human Behaviour-Change Project*, which pioneered the integration of machine learning and ontology-informed modeling to synthesize and interpret behavior change intervention evidence. ^41^ James Thomas at the EPPI Centre has developed AI tools and living evidence platforms for automating literature screening and synthesis, contributing extensively to methodological innovation in systematic review automation. ^55^ Our team also includes researchers with expertise in applying AI to automate the extraction of implementation-relevant data such as barriers, facilitators, and strategies from qualitative and quantitative sources (e.g., Chan, Taylor). ^40^ Several team members (e.g., Abbasgholizadeh-Rahimi, Légaré, Graham, Welch, Presseau, Straus) are leading experts in informatics, data science, and implementation evaluation, and bring deep experience in using implementation science frameworks (e.g., CFIR, KTA, RE-AIM, NASSS) in both high-income and resource-limited settings. Several investigators (e.g., Fontaine, Welch, Straus, Graham, Taylor) have led large-scale knowledge syntheses, scoping reviews, and methodological studies that shape the implementation science evidence base. Others (e.g., Graham, Powell, Michie, Presseau) have co-developed or refined key frameworks and taxonomies for implementation strategies and behavior change techniques that underpin this review’s analytic structure. Together, our team has the methodological, technical, and domain-specific expertise to conduct a comprehensive, high-quality, and policy-relevant review that will support the responsible and equitable integration of AI in implementation research and practice.

### Eligibility criteria

The eligibility criteria for this scoping review are designed to align with JBI’s Population, Concept, and Context (PCC). ^51^ Studies and reports will be included if they meet the following eligibility criteria.

**Population**

We will consider studies involving any individuals or organizations actively engaged in implementation research and practice. This includes researchers, practitioners, administrators, and other stakeholders who focus on any of the KTA key steps including, but not limited to AI-driven synthesis of implementation evidence, identifying and prioritizing the problem, adapting evidence to local contexts, assessing barriers and facilitators, selecting, tailoring and operationalizing implementation strategies, monitoring outcomes and fidelity, evaluating impact on practice and population, and ultimately sustaining and scaling knowledge use. We will include studies only if the focus is on using AI to facilitate these steps, rather than on AI as the intervention itself.

**Concept**

The concept for inclusion requires that studies or reports specifically address the use of AI to support implementation research or practice. As presented in Table 1, we will consider studies or reports that describe AI-assisted approaches encompassing ML, DL, NLP, LLMs or other technologies across all steps of the KTA Model. We will also include cross-cutting features of AI technologies, such as data analysis or predictive modeling, that may not align precisely with a single KTA step but facilitate implementation activities throughout the cycle.

**Table 1.** Potential applications of AI to implementation research and practice activities.

Step along the KTA model	Definition
**1. Synthesize Implementation Evidence**	Using AI to automate the identification, extraction, and synthesis of implementation evidence (e.g., barriers, facilitators, or implementation strategies) from large datasets, including research articles, guidelines, and reports. This might include linked decision support tools.
**2. Identify & Prioritize the Problem, Select Evidence-Based Intervention (EBI)**	Using AI-driven approaches to identify evidence–practice gaps, emerging clinical needs, or high-impact problems by analyzing large volumes of data (e.g., research articles, electronic health records). This may include anomaly detection, trend detection, and predictive modeling to highlight priority issues.
**3. Adapt or Tailor EBI**	Using AI to adapt/tailor evidence-based guidelines, tools, or interventions for local contexts, including automated translations, reading-level adjustments, or context-sensitive content generation.
**4. Assess Barriers & Facilitators (Contextual Analysis)**	Using AI-powered data analysis (e.g., sentiment analysis, topic modeling) to identify challenges, constraints, and enablers within an organization or community. These insights can stem from qualitative sources (interviews, focus groups) and quantitative data.
**5. Select, Tailor and Operationalized Implementation Strategies**	Using AI-based decision support systems or recommendation engines to map identified barriers and facilitators to evidence-based implementation strategies. Deploying AI tools to manage, schedule, or coordinate resources and personnel during rollout.
**6. Monitor EBI Implementation**	Using AI to assess whether an intervention is delivered as intended (fidelity), measure its uptake (reach, acceptability), and collect its real-time performance data. This may include applying behavioural analytics to data sources such as tracking logs or digital footprints.
**7. Evaluate Impact of EBI on Practice & Population**	Using AI to assess intervention impact on clinical, service, or implementation outcomes, or clarify which specific elements of the intervention drive effectiveness, enabling more targeted refinements and better resource allocation.
**8. Sustain & Scale EBI**	Using AI to support the long-term embedding and expansion of successful interventions. This may include predictive models that generalize from existing data to new contexts, as well as equity-focused algorithms that detect disparities in reach or outcomes.

We will exclude studies in which AI is purely employed as the primary clinical or public health intervention (e.g., a clinical decision support system used directly for patient diagnosis, an AI-driven therapy tool) without a clear focus on supporting or studying the implementation process itself. We will exclude studies that do not address steps in the KTA Model, or do not articulate outcomes related to implementation processes (e.g., fidelity, adoption, reach) or implementation-related impact (e.g., changes in practice, sustainability). We will exclude documents that do not present empirical evidence or methodological details about the use of AI in supporting implementation (e.g., purely theoretical or opinion-based articles without data or systematic description of AI application).

**Context**

AI applications relevant to various health domains of implementation science, including but not limited to healthcare settings, health policy implementation, and community-based healthcare interventions, will be included.

**Evidence sources**

We will include primary research studies, scoping reviews, systematic reviews, case reports, grey literature, conference abstracts, and policy documents that describe or evaluate AI applications in implementation science. Both quantitative and qualitative studies are eligible, as well as mixed-methods studies that examine AI’s impact on implementation processes or outcomes.

**Language and date restrictions**

Studies and reports will be limited to those available in English and French, and published within the last 15 years, given the recent advancements in AI technologies.

### Literature search

**Information sources**

The bibliographical databases to be searched include CINAHL, Embase, IEEExplore, MEDLINE, PsycINFO and Web of Science. Furthermore, we will hand-search relevant journals and conference proceedings to identify additional records. Examples of journals may include: *BMJ Quality & Safety*, *Implementation Science*, *Implementation Science Communications*, *BMC Health Services Research*, *Implementation research and Practice*, and *Health Research Policy and Systems.* Examples of relevant conferences include EMNLP/ICML. We will screen the reference list of included records to identify additional records. We will also search relevant pre-print servers (e.g., arXiv). Finally, we will identify a limited number of ‘core papers’ and perform a citation search.

**Search strategy**

Our search strategy has been developed in collaboration with a research librarian (RL) and AI specialists (JC, JH, SAR). It uses a combination of MeSH terms and search terms structured around two core concepts: “artificial intelligence technologies” AND “implementation science activities.” The Medline search strategy is presented in Table 2.

**Table 2.** Medline search strategy.

#	Search terms	Results
**Medline search strategy (Implementation science keywords)**		
1	exp Artificial Intelligence/ OR exp Natural Language Processing/OR (artificial intelligence or Natural Language Processing or “ai” or “machine learning” or “deep learning” or “neural network” or “large language model” or “generative model*” or LLM* or “transformer model*” or “language model*” or “generative AI” or “foundation model*” or “predictive model*” or “supervised learning” or “unsupervised learning” or “reinforcement learning” or “expert system*” or “pattern recognition” or “text mining” or “literature mining” or “evidence extraction” or “automated review” or “sentiment analysis” or “topic modeling” or “text classification” or “counterfactual analysis” or “scenario analysis” or “bias detection” or “ChatGPT” or “GPT-” or BERT or RoBERTa or Gemma or LLaMA).af.	
2	exp Translational Research, Biomedical/ or Quality Improvement/ or Health Services Research/ or Learning Health System/ or exp Organizational Innovation/ or exp Models, Theoretical/ or exp Implementation Science/ or exp “Diffusion of Innovation”/or (Organizational Innovation or Translational Research or diffusion of innovation or “implementation science” or “implementation research” or implementation practice* or “quality improvement” or “improvement science” or “learning health system” or “learning healthcare system” or implementation strateg* or implementation process* or “knowledge translation” or “knowledge to action” or “intervention uptake” or “intervention adoption” or intervention outcome* or “program implementation” or “behavior change” or “behaviour change” or “dissemination and implementation” or “practice change” or "real-world implementation” or “translation of evidence” or framework* or model or models or theory or theories or implementation outcome*).af. or ((implementation or intervention) adj30 (fidelity or “scale-up” or acceptability or feasibility or penetration or adoption or appropriateness or implementability or adoptability or sustainability or spread)).ab,ti,hw,kf,sh. or (implementation adj30 (feasibility or automation or sustain* or barrier* or enabler* or facilitator*)).ab,ti,hw,kf,sh.	
3	“evidence based”.af. or exp Evidence-Based Medicine/	
4	(application or applying or using or utilization or utilizing or leveraging or leverage or “role of” or “impact of” or integrating or integrate or integrated or improving or enhancing or optimizing or optimize or optimization or facilitating or accelerating or supporting or streamlining or streamlined or automating or automate or predicting or personalizing or personalized).ab,ti. adj10 (implementation or translation or knowledge or diffusion).ti,ab.	
5	1 and 2 and 3 and 4	329
6	Limit to 2010-present	309
**Medline search strategy (Implementation science journals)**		
1	exp Artificial Intelligence/ OR exp Natural Language Processing/OR (artificial intelligence or Natural Language Processing or “ai” or “machine learning” or “deep learning” or “neural network” or “large language model” or “generative model*” or LLM* or “transformer model*” or “language model*” or “generative AI” or “foundation model*” or “predictive model*” or “supervised learning” or “unsupervised learning” or “reinforcement learning” or “expert system*” or “pattern recognition” or “text mining” or “literature mining” or “evidence extraction” or “automated review” or “sentiment analysis” or “topic modeling” or “text classification” or “counterfactual analysis” or “scenario analysis” or “bias detection” or “ChatGPT” or “GPT-” or BERT or RoBERTa or Gemma or LLaMA).af.	
2	("Implementation Science” or “JBI Evidence Implementation” or “Global Implementation Research and Applications” or “Translational Behavioral Medicine” or “Implementation Science Communications” or “Implementation Research and Practice”).jn.	
3	1 AND 2	
4	limit 3 to yr="2010 -Current"	
5	After Removing duplicates in Endnote from previous search	124 left
**Medline search strategy (Other journals)**		
1	exp Artificial Intelligence/ OR exp Natural Language Processing/OR (artificial intelligence or Natural Language Processing or “ai” or “machine learning” or “deep learning” or “neural network” or “large language model” or “generative model*” or LLM* or “transformer model*” or “language model*” or “generative AI” or “foundation model*” or “predictive model*” or “supervised learning” or “unsupervised learning” or “reinforcement learning” or “expert system*” or “pattern recognition” or “text mining” or “literature mining” or “evidence extraction” or “automated review” or “sentiment analysis” or “topic modeling” or “text classification” or “counterfactual analysis” or “scenario analysis” or “bias detection” or “ChatGPT” or “GPT-” or BERT or RoBERTa or Gemma or LLaMA).af.	
2	("BMC Health Services Research” or “BMJ Quality & Safety” or “Health Research Policy and Systems” or “Annual of Review of Public Health” or “American Journal of Public Health” or “American Journal of Preventive Medicine”).jn.	
3	1 AND 2	
4	limit 3 to yr="2010 -Current"	
5	After Removing duplicates in Endnote from previous search	837 left

**Update frequency**

We will perform search updates every six months to identify newly published peer-reviewed studies, preprints, and grey literature. The frequency may be adjusted based on the volume of newly identified records and available team capacity. Each update cycle will include re-running the database searches, screening, full-text review, and data extraction following the same procedures as the original review.

**Versioning and reporting**

All updates will be tracked and versioned. New findings and changes in key concepts, classifications, or gaps will be reported in a cumulative manner and noted clearly in any published outputs. A dedicated section will be added to the online supplementary materials or review platform (if hosted) to indicate the date of the last update and planned date for the next update. We will consider developing an interactive database and data visualization if resources allow it.

**Team roles and governance**

GF, NT and other team members will be responsible for overseeing the living component of the review. Team meetings will be scheduled at each update point to discuss inclusion of new evidence and refine the approach if needed.

**Triggers for substantive review revision**

A full update of the review (including potential resubmission for publication) will be triggered if: (i) there is a critical mass of new studies (e.g., >20% increase in included records); (ii) stakeholder priorities or core concepts in implementation science shift meaningfully; (iii) major AI-related methodological and technological breakthroughs occur; or (iv) regulatory/policy developments occur (e.g., WHO guidance on AI in public health).

### Source of evidence selection

After completing the search, all identified citations will be compiled and uploaded into Covidence, where duplicates will be removed. Two independent reviewers will screen all titles and abstracts independently to determine eligibility based on the inclusion criteria for the review. The full texts of selected citations will then be thoroughly evaluated against the inclusion criteria by two independent reviewers. Any disagreements during the selection process will be resolved through discussion or, if necessary, by consulting an additional reviewer. The search results and study selection process will be fully detailed in the final scoping review and displayed in a PRISMA-ScR flow diagram. ^53^

### Data extraction

We will systematically extract detailed information from each included study or other relevant sources to address the review objectives. A structured data extraction form will be developed and piloted to ensure consistent data collection across studies covering article characteristics, evaluation methodology, AI application, comparator, outcomes, adverse effects, and research gaps. Two independent reviewers will conduct the data extraction, after a calibration exercise on 10 articles. Any inconsistencies will be discussed and resolved, and the extraction guide adapted as needed.

**Article characteristics**

We will first extract key article characteristics, including article type, author(s), health and social care categories, year of publication, country of origin, population(s) and setting.

**Evaluation methodology**

We will extract the evaluation methodologies used to assess AI-supported implementation science activities, including quantitative (e.g., RCTs, observational, simulation), qualitative (e.g., interviews, focus groups), and mixed-methods approaches. We will also capture human-centered evaluations (e.g., usability testing), AI-specific techniques (e.g., cross-validation, explainability assessments), and use of implementation science frameworks (e.g., RE-AIM, CFIR, NASSS). This will inform a taxonomy of evaluation approaches for AI-enabled implementation science activities.

**AI application**

We will extract detailed information on the AI application. We will use established classifications, such as the *Living Map of Generative LLM-Based Tools for Health and Social Care Applications* ^55^ (developed by JT), to guide data collection across the following dimensions:

(i) Application class (es) (e.g., clinical service delivery, public health, or policy implementation);
(ii) AI technology (e.g., knowledge-based or rules-based AI using explicit knowledge representation and reasoning, traditional [“shallow”] machine learning [e.g., logistic regression, SVMs], deep learning but without any generative capability or transfer learning, transfer learning including fine-tuning pre-trained foundation models, generative AI, potentially with in-context learning, but without significant additional fine-tuning);
(iii) Model(s), ontology(ies), platform(s) or tool(s) used (e.g., decision trees, neural networks, Bayesian networks; GPT-4, BERT);
(iv) Training datasets and training design used across model(s);
(v) Mode(s) of model use;
(vi) Model version;
(vii) Maturity level of the AI application (e.g., MSc thesis prototype vs. commercial tool);
(viii) Degree of testing and deployment of AI application;
(ix) Implementation science task type(s) of the AI application, categorized according to core implementation science activities (e.g., as per the KTA Model in Table 1).

**Comparator**

For studies that include a comparator, we will document the type of comparator used (e.g., human researcher or clinician, standard manual process, non-AI technology, or another AI model), and the function being compared (e.g., diagnostic accuracy, decision-making, time to task completion). This information will help contextualize the performance of AI applications and support future benchmarking efforts.

**Outcomes**

We will extract information on performance indicators and outcome types reported in relation to AI-supported implementation science activities. While we will not extract specific effect sizes or interpret the direction of effects, we aim to comprehensively map and categorize the types of outcomes assessed across studies. These outcomes will inform the development of a future taxonomy for evaluating AI in implementation science. Outcome types may include:

(i) Time-related outcomes: Time to complete tasks, time to implementation, delays, etc.
(ii) Cost-related outcomes: Development costs, operational costs, cost-effectiveness metrics, etc.
(iii) Accuracy: Concordance with gold standard or expert judgement, reduction in errors, etc.
(iv) Task-specific technical metrics, depending on the nature of the AI model:
  a. Classification tasks: Precision, recall, F1-score, AUC-ROC, specificity, sensitivity.
  b. Regression tasks: Mean Absolute Error (MAE), Mean Squared Error (MSE), Root Mean Squared Error (RMSE), R ^2^.
  c. Generative tasks: BLEU, ROUGE, METEOR, GLEU, perplexity scores.
  d. Ranking or recommendation tasks: NDCG, MAP, MRR.
  e. Human factors: Usability, user satisfaction, acceptability, and trust in the system.
(v) Implementation-specific outcomes: Adoption, fidelity, reach, sustainability, feasibility, etc.
(vi) Equity-related outcomes: Disparities in performance across subgroups, etc.
(vii) Clinical and health system outcomes (where relevant): Patient satisfaction, clinical workflow improvements, adherence to guidelines, or patient safety markers.

**Adverse effects**

We will extract any reported or potential adverse effects or unintended consequences associated with AI use in implementation science. This includes clinical or patient harms, such as treatment errors; systemic issues like increased clinician workload, workflow disruptions, or exacerbation of disparities caused by existing biases in training data; and AI-specific risks, including algorithmic drift, hallucinations in generative models, or overreliance on automated systems. We will also document user-level effects such as reduced trust, cognitive overload, decision fatigue, or de-skilling of professionals. All identified harms will be classified and mapped to support future risk assessment and mitigation efforts.

**Research gaps**

Finally, we will identify both explicitly stated and inferred research gaps to enhance the role of AI in implementation science. These may include gaps in knowledge or evidence related to AI’s effectiveness, scalability, or sustainability in implementation contexts; underexplored domains of application (e.g., underrepresented populations, low-resource settings), methodological gaps (e.g., lack of robust evaluation, absence of longitudinal studies), and conceptual or theoretical gaps (e.g., insufficient use of implementation science frameworks, lack of interdisciplinary integration). We will also capture recommendations made by study authors for future AI development, evaluation, or use in implementation science, needs for standards, reporting guidelines, or regulatory frameworks to support responsible AI use. These gaps will inform a future research agenda and highlight opportunities to enhance the value and equity of AI in implementation science.

### Data analysis and synthesis

We will conduct a structured analysis of included studies to address the review’s objectives. First, we will generate a descriptive summary capturing key characteristics such as publication year, country of origin, study design, setting, population, and area of application. This will allow us to identify trends in how AI is being used within implementation science. AI applications will be categorized by the implementation activity they support, the type of technology used, the specific tools or models described, and their level of maturity. Outcomes will be grouped and summarized based on their relevance to performance, implementation, human factors, equity, and system-level impact. We will describe how outcomes are measured and reported, but not interpret effect sizes. Findings will be integrated into a narrative synthesis that links AI applications, implementation activities, outcomes, and research gaps.

## Conclusion

This living scoping review will offer a comprehensive overview of how AI is being applied across the spectrum of implementation science activities. It will map the current landscape, synthesize reported outcomes, and identify key research gaps. The findings will serve as a foundation for advancing the responsible and equitable integration of AI in implementation research and practice, with the potential to accelerate the adoption, scale-up, and sustainability of evidence-based interventions. Target audiences include implementation scientists, applied researchers, funding agencies, computer scientists seeking to engage with real-world challenges, implementation practitioners, and policymakers.

## Disclosures

ChatGPT 4o (OpenAI, 2025) was used to enhance the coherence and readability of some sections of this manuscript. The authors have reviewed all sections of the article and take full responsibility for its contents.

## Data Availability

This article type (protocol) does not require data.

## References

[1] Glasziou P Straus S Brownlee S : Evidence for underuse of effective medical services around the world. *Lancet.* Jul 8 2017;390(10090):169–177. 10.1016/S0140-6736(16)30946-1 28077232

[2] Brownlee S Chalkidou K Doust J : Evidence for overuse of medical services around the world. *Lancet.* Mar 5 2017;390:156–168. 10.1016/S0140-6736(16)32585-5 28077234 PMC5708862

[3] Eccles MP Mittman BS : Welcome to Implementation Science. *Implement. Sci.* 2006;1:1. 10.1186/1748-5908-1-1

[4] Fontaine G Taylor N Bruneau J : The urgent need for implementation science to achieve hepatitis C elimination. *Lancet Gastroenterol. Hepatol.* Mar 4 2025;10:498–502. 10.1016/S2468-1253(25)00050-0 40054488

[5] Sergerie-Richard S Goulet MH Dumais A : De-implementation to reduce coercive practices in mental health care. *Lancet Psychiatry.* Jul 2024;11(7):498–499. 10.1016/S2215-0366(24)00144-5 38879271

[6] Mustanski B Smith JD Keiser B : Supporting the Growth of Domestic HIV Implementation Research in the United States Through Coordination, Consultation, and Collaboration: How We Got Here and Where We Are Headed. *J. Acquir. Immune Defic. Syndr.* Jul 1 2022;90(S1):S1–S8. 10.1097/QAI.0000000000002959 35703749 PMC9643076

[7] Oh AY Emmons KM Brownson RC : Speeding implementation in cancer: The National Cancer Institute’s Implementation Science Centers in Cancer Control. *J. Natl. Cancer Inst.* Feb 8 2023;115(2):131–138. 10.1093/jnci/djac198 36315080 PMC9905952

[8] Donohue JF Elborn JS Lansberg P : Bridging the “Know-Do” Gaps in Five Non-Communicable Diseases Using a Common Framework Driven by Implementation Science. *J. Healthc. Leadersh.* 2023;15:103–119. 10.2147/JHL.S394088 37416849 PMC10320809

[9] Sartor C Hussian M : Mental health implementation science: integrating lived experience expertise. *Lancet Psychiatry.* May 2024;11(5):321–322. 10.1016/S2215-0366(24)00092-0 38552661

[10] Fontaine G Mooney M Porat-Dahlerbruch J : Advancing the selection of implementation science theories, models, and frameworks: A scoping review and the development of the SELECT-IT meta-framework. *Implement. Sci.* 2025;20:24. 10.1186/s13012-025-01436-5 40437531 PMC12117738

[11] Patey AM Fontaine G Francis JJ : Healthcare professional behaviour: health impact, prevalence of evidence-based behaviours, correlates and interventions. *Psychol. Health.* Jun 2023;38(6):766–794. 10.1080/08870446.2022.2100887 35839082

[12] Sturke R Harmston C Simonds RJ : A multi-disciplinary approach to implementation science: the NIH-PEPFAR PMTCT implementation science alliance. *J. Acquir. Immune Defic. Syndr.* Nov 1 2014;67 Suppl 2:S163–S167. 10.1097/QAI.0000000000000323 25310124

[13] Harvey G Rycroft-Malone J Seers K : Connecting the science and practice of implementation - applying the lens of context to inform study design in implementation research. *Front Health Serv.* 2023;3:1162762. 10.3389/frhs.2023.1162762 37484830 PMC10361069

[14] Rapport F Smith J Hutchinson K : Too much theory and not enough practice? The challenge of implementation science application in healthcare practice. *J. Eval. Clin. Pract.* Dec 2022;28(6):991–1002. 10.1111/jep.13600 34268832

[15] Morrow A Tyedmers E Debono D : Trials and tribulations: a qualitative exploration of researcher perspectives on navigating the challenges of health system implementation research. *BMJ Open.* Jan 15 2025;15(1):e087926. 10.1136/bmjopen-2024-087926 39819940 PMC11784370

[16] Baumann AA Cabassa LJ : Reframing implementation science to address inequities in healthcare delivery. *BMC Health Serv. Res.* Mar 12 2020;20(1):190. 10.1186/s12913-020-4975-3 32164706 PMC7069050

[17] Beidas RS Dorsey S Lewis CC : Promises and pitfalls in implementation science from the perspective of US-based researchers: learning from a pre-mortem. *Implement. Sci.* Aug 13 2022;17(1):55. 10.1186/s13012-022-01226-3 35964095 PMC9375077

[18] Rashid AB Kausik MDAK : AI revolutionizing industries worldwide: A comprehensive overview of its diverse applications. *Hybrid Advances.* 2024;7:100277. 10.1016/j.hybadv.2024.100277

[19] Haug CJ Drazen JM : Artificial Intelligence and Machine Learning in Clinical Medicine, 2023. *N. Engl. J. Med.* Mar 30 2023;388(13):1201–1208. 10.1056/NEJMra2302038 36988595

[20] Fontaine G Cossette S Maheu-Cadotte MA : Efficacy of adaptive e-learning for health professionals and students: a systematic review and meta-analysis. *BMJ Open.* Aug 28 2019;9(8):e025252. 10.1136/bmjopen-2018-025252 31467045 PMC6719835

[21] OpenAI: GPT-4 Technical Report. 2024.

[22] Hugo Touvron TL Izacard G Martinet X : LLaMA: Open and Efficient Foundation Language Models. 2023.

[23] Gemma Team: Gemma: Open Models Based on Gemini Research and Technology. 2024.

[24] Ahsan MM Luna SA Siddique Z : Machine-Learning-Based Disease Diagnosis: A Comprehensive Review. *Healthcare (Basel).* Mar 15 2022;10(3). 10.3390/healthcare10030541 35327018 PMC8950225

[25] Myszczynska MA Ojamies PN Lacoste AMB : Applications of machine learning to diagnosis and treatment of neurodegenerative diseases. *Nat. Rev. Neurol.* Aug 2020;16(8):440–456. 10.1038/s41582-020-0377-8 32669685

[26] Kim HE Kim HH Han BK : Changes in cancer detection and false-positive recall in mammography using artificial intelligence: a retrospective, multireader study. *Lancet Digit Health.* Mar 2020;2(3):e138–e148. 10.1016/S2589-7500(20)30003-0 33334578

[27] McKinney SM Sieniek M Godbole V : International evaluation of an AI system for breast cancer screening. *Nature.* Jan 2020;577(7788):89–94. 10.1038/s41586-019-1799-6 31894144

[28] Abubaker Bagabir S Ibrahim NK Abubaker Bagabir H : Covid-19 and Artificial Intelligence: Genome sequencing, drug development and vaccine discovery. *J. Infect. Public Health.* Feb 2022;15(2):289–296. 10.1016/j.jiph.2022.01.011 35078755 PMC8767913

[29] Guedj M Swindle J Hamon A : Industrializing AI-powered drug discovery: lessons learned from the Patrimony computing platform. *Expert Opin. Drug Discov.* Aug 2022;17(8):815–824. 10.1080/17460441.2022.2095368 35786124

[30] Ahmed F Kang IS Kim KH : Drug repurposing for viral cancers: A paradigm of machine learning, deep learning, and virtual screening-based approaches. *J. Med. Virol.* Apr 2023;95(4):e28693. 10.1002/jmv.28693 36946499

[31] Nelson KM Chang ET Zulman DM : Using Predictive Analytics to Guide Patient Care and Research in a National Health System. *J. Gen. Intern. Med.* Aug 2019;34(8):1379–1380. 10.1007/s11606-019-04961-4 31011959 PMC6667597

[32] Curtis RG Bartel B Ferguson T : Improving User Experience of Virtual Health Assistants: Scoping Review. *J. Med. Internet Res.* Dec 21 2021;23(12):e31737. 10.2196/31737 34931997 PMC8734926

[33] Graham S Depp C Lee EE : Artificial Intelligence for Mental Health and Mental Illnesses: an Overview. *Curr. Psychiatry Rep.* Nov 7 2019;21(11):116. 10.1007/s11920-019-1094-0 31701320 PMC7274446

[34] Lee P Bubeck S Petro J : Benefits, Limits, and Risks of GPT-4 as an AI Chatbot for Medicine. *N. Engl. J. Med.* Mar 30 2023;388(13):1233–1239. 10.1056/NEJMsr2214184 36988602

[35] Safranek CW Sidamon-Eristoff AE Gilson A : The Role of Large Language Models in Medical Education: Applications and Implications. *JMIR Med Educ.* Aug 14 2023;9:e50945. 10.2196/50945 37578830 PMC10463084

[36] Aydin S Karabacak M Vlachos V : Large language models in patient education: a scoping review of applications in medicine. *Front Med (Lausanne).* 2024;11:1477898. 10.3389/fmed.2024.1477898 39534227 PMC11554522

[37] Trinkley KE An R Maw AM : Leveraging artificial intelligence to advance implementation science: potential opportunities and cautions. *Implement. Sci.* Feb 21 2024;19(1):17. 10.1186/s13012-024-01346-y 38383393 PMC10880216

[38] Graham ID Logan J Harrison MB : Lost in knowledge translation: time for a map? *J. Contin. Educ. Heal. Prof.* Winter 2006;26(1):13–24. 10.1002/chp.47 16557505

[39] Michie S Thomas J Johnston M : The Human Behaviour-Change Project: harnessing the power of artificial intelligence and machine learning for evidence synthesis and interpretation. *Implement. Sci.* Oct 18 2017;12(1):121. 10.1186/s13012-017-0641-5 29047393 PMC5648456

[40] Chan J Lin F Tran M : Systematic and AI-assisted curation of knowledge ontology to support rapid learning in implementation science. *Presented at: Society for Implementation Research Collaboration (SIRC) Conference.* Denver, CO, United States:2024.

[41] West R Bonin F Thomas J : Using machine learning to extract information and predict outcomes from reports of randomised trials of smoking cessation interventions in the Human Behaviour-Change Project. *Wellcome Open Research.* 2024;8:452. 10.12688/wellcomeopenres.20000.2 38779058 PMC11109593

[42] Hastings J Glauer M West R : Predicting outcomes of smoking cessation interventions in novel scenarios using ontology-informed, interpretable machine learning. *Wellcome Open Research.* 2023;8. 10.12688/wellcomeopenres.20012.1 PMC12603517 41230249

[43] Hussain Z Sheikh Z Tahir A : Artificial Intelligence-Enabled Social Media Analysis for Pharmacovigilance of COVID-19 Vaccinations in the United Kingdom: Observational Study. *JMIR Public Health Surveill.* May 27 2022;8(5):e32543. 10.2196/32543 35144240 PMC9150729

[44] Guetterman TC Chang T DeJonckheere M : Augmenting Qualitative Text Analysis with Natural Language Processing: Methodological Study. *J. Med. Internet Res.* Jun 29 2018;20(6):e231. 10.2196/jmir.9702 29959110 PMC6045788

[45] Spinoso-Di Piano C Rahimi S Cheung J : *Qualitative Code Suggestion: A Human-Centric Approach to Qualitative Coding.* Association for Computational Linguistics;2023;14887–14909.

[46] Chustecki M : Benefits and Risks of AI in Health Care: Narrative Review. *Interact. J. Med. Res.* Nov 18 2024;13:e53616. 10.2196/53616 39556817 PMC11612599

[47] Dankwa-Mullan I : Health Equity and Ethical Considerations in Using Artificial Intelligence in Public Health and Medicine. *Prev. Chronic Dis.* Aug 22 2024;21:E64. 10.5888/pcd21.240245 39173183 PMC11364282

[48] Goktas P Grzybowski A : Shaping the Future of Healthcare: Ethical Clinical Challenges and Pathways to Trustworthy AI. *J. Clin. Med.* Feb 27 2025;14(5). 10.3390/jcm14051605 40095575 PMC11900311

[49] Katirai A : The Environmental Costs of Artificial Intelligence for Healthcare. *Asian Bioeth Rev.* 2024;16(3):527–538. 10.1007/s41649-024-00295-4 39022383 PMC11250743

[50] Pollock DK Khalil H Evans C : The role of scoping reviews in guideline development. *J. Clin. Epidemiol.* 2024;169:111301. 10.1016/j.jclinepi.2024.111301 38423402

[51] Peters MDJ Godfrey C McInerney P : Chapter 11: Scoping reviews (2020 version). Aromataris E Munn Z , editors. *Joanna Briggs Institute (JBI) manual for evidence synthesis.* JBI;2020.

[52] Cochrane: Guidance for the production and publication of Cochrane living systematic reviews: Cochrane Reviews in living mode. 2019. Reference Source

[53] Tricco AC Lillie E Zarin W : PRISMA Extension for Scoping Reviews (PRISMA-ScR): Checklist and Explanation. *Ann. Intern. Med.* Oct 2 2018;169(7):467–473. 10.7326/M18-0850 30178033

[54] Akl EA Khabsa J Iannizzi C : Extension of the PRISMA 2020 statement for living systematic reviews (PRISMA-LSR): checklist and explanation. *BMJ.* Nov 19 2024;387:e079183. 10.1136/bmj-2024-079183 39562017 PMC12036629

[55] Shemilt IHG Khouja C Raine G : *Generative large language models for health and social care applications: a living map of research.* EPPI Centre;2024.

