# Supplementary material for: Tracking the Evolving Role of Artificial Intelligence in Implementation Science: Protocol for a Living Scoping Review of Applications, Evaluation Approaches and Outcomes
Source: F1000Res. 2026 Feb 12;14:1135. Originally published 2025 Oct 17. [Version 2] doi: 10.12688/f1000research.171774.2 (PMC12988357; doi:10.12688/f1000research.171774.2)
Supplement: Peer review history

# Reviewer report: Reviewer response for version 2

**DOI:** 10.5256/f1000research.192327.r436845

**Published:** 2026-08-14

**Authors:** Omid Panahi

**Affiliations:** 1 Centro Escolar University, Manila, Metro Manila, Philippines

This article presents a well-developed protocol for a living scoping review that aims to track and synthesize the evolving use of artificial intelligence (AI) in implementation science. The proposed review seeks to map AI applications across implementation research and practice, identify evaluation approaches and outcomes, examine risks and unintended consequences, and highlight future research opportunities. Given the rapid pace of AI development and its growing relevance to implementation science, the rationale for conducting a living scoping review is compelling and well justified.

 The objectives are clearly stated and aligned with the proposed methodology. The authors appropriately employ the Joanna Briggs Institute (JBI) methodology for scoping reviews and Cochrane guidance for living reviews. The use of the Knowledge-to-Action (KTA) framework provides a strong conceptual foundation for categorizing AI applications across implementation activities. The inclusion criteria are comprehensive and clearly defined, covering a broad range of evidence sources while maintaining a specific focus on AI applications that support implementation processes rather than AI as the intervention itself.

 The search strategy is extensive and methodologically sound, incorporating multiple bibliographic databases, conference proceedings, grey literature sources, preprint servers, citation tracking, and hand-searching approaches. The protocol also provides sufficient detail regarding study selection, data extraction, update frequency, version control, governance, and synthesis methods, allowing replication by other researchers.

 A particular strength of the protocol is the comprehensive data extraction framework. The planned extraction of information on AI technologies, implementation activities, evaluation methodologies, outcomes, adverse effects, equity considerations, and research gaps will provide a valuable resource for researchers and practitioners. The explicit attention to potential harms, unintended consequences, algorithmic bias, and equity issues demonstrates a balanced and responsible approach to AI evaluation.

 The living review methodology is especially appropriate given the rapidly evolving nature of AI technologies and their applications in healthcare and implementation science. The planned six-month update cycle and predefined triggers for major review updates further strengthen the methodological rigor of the protocol.

 Although no major concerns were identified, future iterations of the review may benefit from further consideration of human oversight and stakeholder involvement in AI-supported implementation activities. Capturing whether and how clinicians, researchers, patients, policymakers, or other stakeholders validate, supervise, or interact with AI-generated outputs could provide additional insights into responsible AI deployment and trustworthiness. Similarly, documenting approaches used to address equity and bias in implementation settings may further strengthen the review's contribution to the field.

 Overall, the rationale is clearly described, the study design is appropriate, and sufficient methodological details are provided to allow replication. As this is a protocol article, the dataset question is not applicable. The manuscript is scientifically sound, methodologically rigorous, and addresses a highly relevant and rapidly emerging area of research. I support its indexing and believe the resulting review will provide a valuable resource for implementation scientists, healthcare researchers, policymakers, and practitioners interested in the responsible integration of AI into implementation science.

Is the study design appropriate for the research question?

Yes

Is the rationale for, and objectives of, the study clearly described?

Yes

Are sufficient details of the methods provided to allow replication by others?

Yes

Are the datasets clearly presented in a useable and accessible format?

No

Reviewer Expertise:

Implementation Science; Artificial Intelligence in Healthcare; Dental Public Health; Evidence Synthesis and Systematic Reviews; Health Services Research; Digital Health; Knowledge Translation; Machine Learning Applications in Healthcare.

I confirm that I have read this submission and believe that I have an appropriate level of expertise to confirm that it is of an acceptable scientific standard.

# Reviewer report: Reviewer response for version 2

**DOI:** 10.5256/f1000research.192327.r462869

**Published:** 2026-03-13

**Authors:** James L David

**Affiliations:** 1 Columbia University, New York, USA

Overall, this is a timely and important review, and I was so happy to be able to review it. I will be revisiting it to check for any updates. Your choice regarding the necessity of a living review is well-supported by the protocol itself, which notes that AI technologies are evolving at an unprecedented pace. The living review might even add some order to how things evolve. Great job and you have a wonderful team assembled. I have no reservations about the Indexing of this review and its approach, just some suggestions of the inclusion of stakeholder voice (see below).

 Specifically, this protocol for a living scoping review is exceptionally timely. From the article, I understood the rapid proliferation of AI-powered tools and how they are fundamentally altering the landscape of implementation science. You all really described well the creation of novel opportunities for evidence synthesis and analysis that were previously inaccessible due to resource constraints. A living approach is particularly appropriate here, as it ensures the evidence base remains current in a field where traditional static reviews would quickly become obsolete.

 The interdisciplinary team is clearly well-equipped to undertake this work, bringing together internationally recognized expertise in implementation science, AI, and behavioral science. Their collective experience with key frameworks like the Knowledge-to-Action (KTA) Model and the Human Behaviour-Change Project (HBCP) provides a robust foundation for deciphering complex results and providing high-quality, relevant insights.

 I would suggest bringing forward Human-in-the-loop (HITL) and Stakeholder themes. These are present in the protocol, but they are tucked into the technical Methods and Eligibility sections. In light of the team's objective to map the responsible and equitable use of AI, there is an opportunity to further strengthen the review by more explicitly tracking the role of the human-in-the-loop when it comes to AI usage in implementation science. While the protocol already includes practitioners and administrators in its population, it would be beneficial to know if the team has considered specifically cataloging the different stages were stakeholders, such as patients or practitioners, vet the data to validate AI-generated results (if at all).

 To go into more specifics about this, there are some additional questions I would be curious about. Could the data extraction phase be used to distinguish between different types of human intervention, such as whether stakeholders are involved in the initial vetting of input data or the final validation of AI-produced strategies? Including a focus on these specific human-focused checks and balances would provide a clearer roadmap for when oversight is most critical to prevent errors like algorithmic bias or hallucinations. Given our collective experience in resource-limited settings, how might this review specifically explore whether these AI tools are closing the implementation gap for marginalized populations or making them worse, and what specific vetting processes are being documented to ensure that equity remains a central pillar of AI-enabled implementation science (again, if at all in these studies and AI integration approaches)?

Is the study design appropriate for the research question?

Yes

Is the rationale for, and objectives of, the study clearly described?

Yes

Are sufficient details of the methods provided to allow replication by others?

Yes

Are the datasets clearly presented in a useable and accessible format?

Yes

Reviewer Expertise:

Implementation Science, Community Engaged Research, AI Integration

I confirm that I have read this submission and believe that I have an appropriate level of expertise to confirm that it is of an acceptable scientific standard.

# Reviewer report: Reviewer response for version 1

**DOI:** 10.5256/f1000research.189425.r425271

**Published:** 2025-11-11

**Authors:** Julia Brasileiro

**Affiliations:** 1 Florida State University, Tallahassee, Florida, USA

In this paper, the authors describe the protocol for a living scoping review aimed at tracking the evolving role of artificial intelligence (AI) in implementation science. Overall, this a strong protocol paper and I am eager to read the findings from this scoping review. The introduction provides a compelling rationale for this study. The overview of AI is helpful as well as the distinction between AI and the methods that power them. The methodology is rigorous, following the Joanna Briggs Institute methodology and Cochrane guidance for living systematic reviews. The Medline search strategy and keywords are robust, developed in collaboration with a research librarian and AI specialists. The planned data extraction is comprehensive, covering characteristics, evaluation methodology, AI application, comparator, outcomes and adverse effects. Including adverse effects is particularly valuable for assessing potential harms associated with AI in the context of implementation – an area that I believe is not yet fully  understood.

 Minor suggested edits for consideration include:

- Spell out the acronym for the KTA model the first time it is used in the main text of the paper to ensure clarity for all readers.
- Consider providing a clearer definition of a “living scoping review.” While the rationale for using the approach is well-argued, a concise definition before providing this rationale would be helpful.  
- In the eligibility criteria section, it may be helpful to more explicitly state that included studies must be in various health domains within implementation science. From the Introduction it was clear that the paper focused on health domains, but it was less clear in the study eligibility criteria section.
- Regarding the use of two independent reviewers for screening abstracts and data extraction, will you report an inter-rater reliability (e.g., percent agreement) between the two reviewers to document consistency and strength of the methodology?  That might be helpful to document if not already planned.

Overall, this study will advance the field and be useful for guiding future implementation science research that incorporates AI.

Is the study design appropriate for the research question?

Yes

Is the rationale for, and objectives of, the study clearly described?

Yes

Are sufficient details of the methods provided to allow replication by others?

Yes

Are the datasets clearly presented in a useable and accessible format?

Not applicable

Reviewer Expertise:

Adolescent health, digital health interventions, implementation science, mixed methods research, systematic and scoping reviews.

I confirm that I have read this submission and believe that I have an appropriate level of expertise to confirm that it is of an acceptable scientific standard.

# Response

**Published:** 2026-01-09

**Authors:** Guillaume Fontaine

**Affiliations:** Ingram School of Nursing, McGill University Faculty of Medicine and Health Sciences, Montreal, Québec, Canada

We thank Reviewer 1 for their thoughtful and encouraging feedback on the protocol. We are grateful for the positive assessment of the study rationale, methodological rigor, search strategy, and the inclusion of adverse effects, which we agree is an important and under-explored aspect of AI use in implementation science. We have addressed each minor comment as follows.

 Comment 1: Spell out the acronym for the KTA model the first time it is used.

**Response:** Thank you for this suggestion. We have now spelled out the Knowledge-to-Action (KTA) model at first mention.

 Comment 2: Provide a clearer definition of a “living scoping review” earlier in the paper.

**Response:** We agree that a concise definition before the rationale strengthens clarity for readers. We have added a brief definition early in the Introduction: “A living scoping review is a scoping review that is kept continually up to date through ongoing, planned searches and screening at regular intervals, with newly eligible evidence incorporated as it becomes available and the review outputs (e.g., tables/figures and narrative synthesis) updated in versioned iterations.”

 Comment 3: Make explicit in the eligibility criteria that included studies must be in various health domains within implementation science.

**Response:** We have revised the eligibility criteria to specify that included studies must be situated in implementation science within health-related domains, consistent with the scope of the review.

 Comment 4: Will you report inter-rater reliability (e.g., percent agreement) for screening and extraction?

**Response:** We appreciate this suggestion and agree that documenting consistency is important. For this living scoping review, we will not report inter-rater reliability statistics. This decision reflects the iterative, team-based nature of a living workflow, in which the reviewer team and procedures may evolve over time as new evidence accrues, making a single reliability metric difficult to interpret and potentially misleading. Title/abstract screening and data extraction will use independent review where feasible, and all disagreements will be tracked and resolved through a structured consensus-based process, including scheduled conflict-resolution meetings, with adjudication by a senior team member when needed.
